# CXCL12/CXCR4 axis induced miR-125b promotes invasion and confers 5-fluorouracil resistance through enhancing autophagy in colorectal cancer

**DOI:** 10.1038/srep42226

**Published:** 2017-02-08

**Authors:** Xinfeng Yu, Wenna Shi, Yuhang Zhang, Xiaohui Wang, Shiyue Sun, Zhiyu Song, Man Liu, Qiao Zeng, Shuxiang Cui, Xianjun Qu

**Affiliations:** 1 Department of Pharmacology, School of Basic Medical Sciences, Capital Medical University, Beijing, China; 2 Department of General Surgery, Xuan Wu Hospital, Capital Medical University, Beijing, China; 3 Beijing Key Laboratory of Environmental Toxicology, Department of Toxicology and Sanitary Chemistry, School of Public Health, Capital Medical University, Beijing, China

## Abstract

The activation of CXCL12/CXCR4 axis is associated with potential progression of cancer, such as invasion, metastasis and chemoresistance. However, the underlying mechanisms of CXCL12/CXCR4 axis and cancer progression have been poorly explored. We hypothesized that miRNAs might be critical downstream mediators of CXCL12/CXCR4 axis involved in cancer invasion and chemoresistance in CRC. In human CRC cells, we found that the activation of CXCL12/CXCR4 axis promoted epithelial-mesenchymal transition (EMT) and concurrent upregulation of miR-125b. Overexpression of miR-125b robustly triggered EMT and cancer invasion, which in turn enhanced the expression of CXCR4. Importantly, the reciprocal positive feedback loop between CXCR4 and miR-125b further activated the Wnt/β-catenin signaling by targeting *Adenomatous polyposis coli* (*APC*) gene. There was a negative correlation of the expression of miR-125b with *APC* mRNA in paired human colorectal tissue specimens. Further experiments indicated a role of miR-125b in conferring 5-fluorouracil (5-FU) resistance in CRC probably through increasing autophagy both *in vitro* and *in vivo*. MiR-125b functions as an important downstream mediator upon the activation of CXCL12/CXCR4 axis that involved in EMT, invasion and 5-FU resistance of CRC. These findings shed a new insight into the role of miR-125b and provide a potential therapeutic target in CRC.

Colorectal cancer (CRC) is among three leading causes of cancer-related death worldwide. Nearly 50% of patients with CRC develop liver metastases which contributed to high mortality of advanced CRC^1^. Recent studies have highlighted the role of chemokines receptor CXCR4 in cancer metastasis^2^,^3^. CXCL12, also known as stromal cell-derived factor-1 (SDF-1), is a ligand for CXCR4. Activation of CXCL12-CXCR4 axis leads to enhanced chemotaxis, transendothelial migration, and invasion^4^. Further evidences suggest a critical role of the CXCL12/CXCR4 axis in tumor initiation, metastatic colonization and resistance to chemotherapy. It has been proposed that activation of oncogenic Wnt/β-catenin and PI3K-Akt signaling, an epithelial-mesenchymal transition (EMT) and regulating cancer stem cells (CSCs) contributed to the initiation, progression and chemoresistance of CRC^5^. The CXCL12/CXCR4 signaling and oncogenic Wnt/β-catenin and PI3K-Akt pathways constitute a positive feedback loop modulating self-renewal capacity and tumorigenesis in mucosal epithelia of colon^6^. The acquisition of EMT program is a critical process for the progression of CRC from local carcinomas to invasive malignancies^7^,^8^. However, the potential targets orchestrated by the CXCL12/CXCR4 metastatic niche are not yet fully understood.

MicroRNAs (miRNAs) have been considered to play important roles in cancer progression by regulating target gene expression at the post-transcriptional level^9^. They emerge as either tumor suppressors or oncomiRs broadly implicated in tumor proliferation, invasion and resistance to therapy^10^. Although dysregulation of miRNAs has been found to be associated with CRC progression^11^, few studies have been investigated concerning miRNAs-mediated EMT and the corresponding signaling pathways. Recently, some studies have shown that the acquisition of EMT in cancer cells is regulated by miRNAs^12^. It is likely that some of the miRNAs might involve in the activation of CXCL12/CXCR4 axis and therefore play an important role in cancer progression and metastasis. Despite the evidence of chemokines mediating miRNA expression and the potential targets in carcinogenesis^13^, the effects of CXCL12/CXCR4 signaling pathway on miRNA expression in CRC have not been fully investigated.

In this study, we found that miR-125b was overexpressed in the tissues of human CRC and the activation of CXCL12/CXCR4 axis was critically associated with the induction of miR-125b in CRC. Furthermore, miR-125b was found to function as a mediator of CXCL12/CXCR4 axis induced invasion and EMT, which in turn enhanced CXCR4 expression, forming a reciprocal positive feedback loop between CXCR4 and miR-125b in CRC. The upregulation of miR-125b further activated the Wnt/β-catenin signaling by targeting *APC* gene and contributed to 5-FU resistance through enhancing cell autophagy. These results shed insights into the role of miR-125b upon activation of CXCL12/CXCR4 axis and provide potential therapeutic targets in CRC.

## Results

### Activation of CXCL12/CXCR4 axis increases miR-125b expression in CRC cancer cells

**Figure 1:**
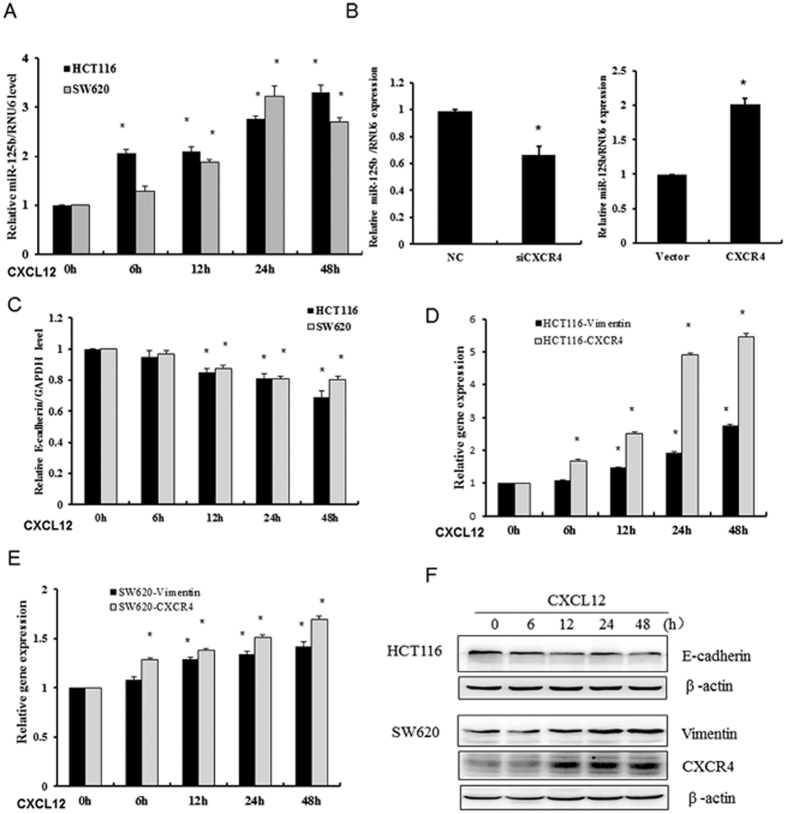
CXCL12 induced upregulation of miR-125b and concurrent EMT in CRC cells. (**A**) HCT116 and SW620 cells were exposed to 100 ng/μl CXCL12 for the indicated time. RT-qPCR was performed to determine the expression of miR-125b. (**B**) HCT116 cells were transfected with 200 nM siRNA of CXCR4 or expression plasmid together with corresponding controls for 48 h. RT-qPCR was performed to determine the expression of miR-125b. (**C**–**E**) The cells were treated as indicated above by CXCL12, the mRNA levels of E-cadherin, vimentin and CXCR4 were determined by RT-qPCR assay. ^*^P < 0.05 *vs.* control. (**F**) The protein level of E-cadherin, vimentin and CXCR4 was examined by Western blot assay.

To explore the roles of miRNAs induced by the activation of CXCL12/CXCR4 axis in CRC, we firstly examined a variety of miRNAs upregulated or downregulated in HCT116 cells overexpressing CXCR4 by sequencing (Supplementary Table 1). MiR-125b was found to be upregulated in response to the stimulation of CXCL12 in both poorly invasive HCT116 cells and highly invasive SW620 cells. RT-qPCR analysis showed an increase of miR-125b expression in a time-dependent manner (0, 6, 12, 24, 48 h) in HCT116 cells as CXCL12/CXCR4 axis was activated by 100 ng/μl CXCL12. SW620 cells also demonstrated a gradually increase of miR-125b and reached a peak at 24 h in response to CXCL12 stimulation (Fig. 1A). Furthermore, cells transfected with CXCR4 robustly increased the level of miR-125b expression in CRC cells, whereas knockdown of CXCR4 reduced miR-125b level compared with control cells (Fig. 1B). To investigate whether activation of CXCL12/CXCR4 axis promotes EMT, we performed the assays of RT-PCR and Western blot to determine the expressions of E-cadherin and vimentin in cancer cells. Results showed that CXCL12 attenuated the expression of E-cadherin time-dependently while enhanced the expression of vimentin as well as CXCR4 (Fig. 1C–F). These results indicated that activation of CXCL12/CXCR4 axis induced miR-125b expression and concurrently promoted EMT in cancer cells.

### Overexpression of miR-125b increased the invasiveness of CRC cells

**Figure 2:**
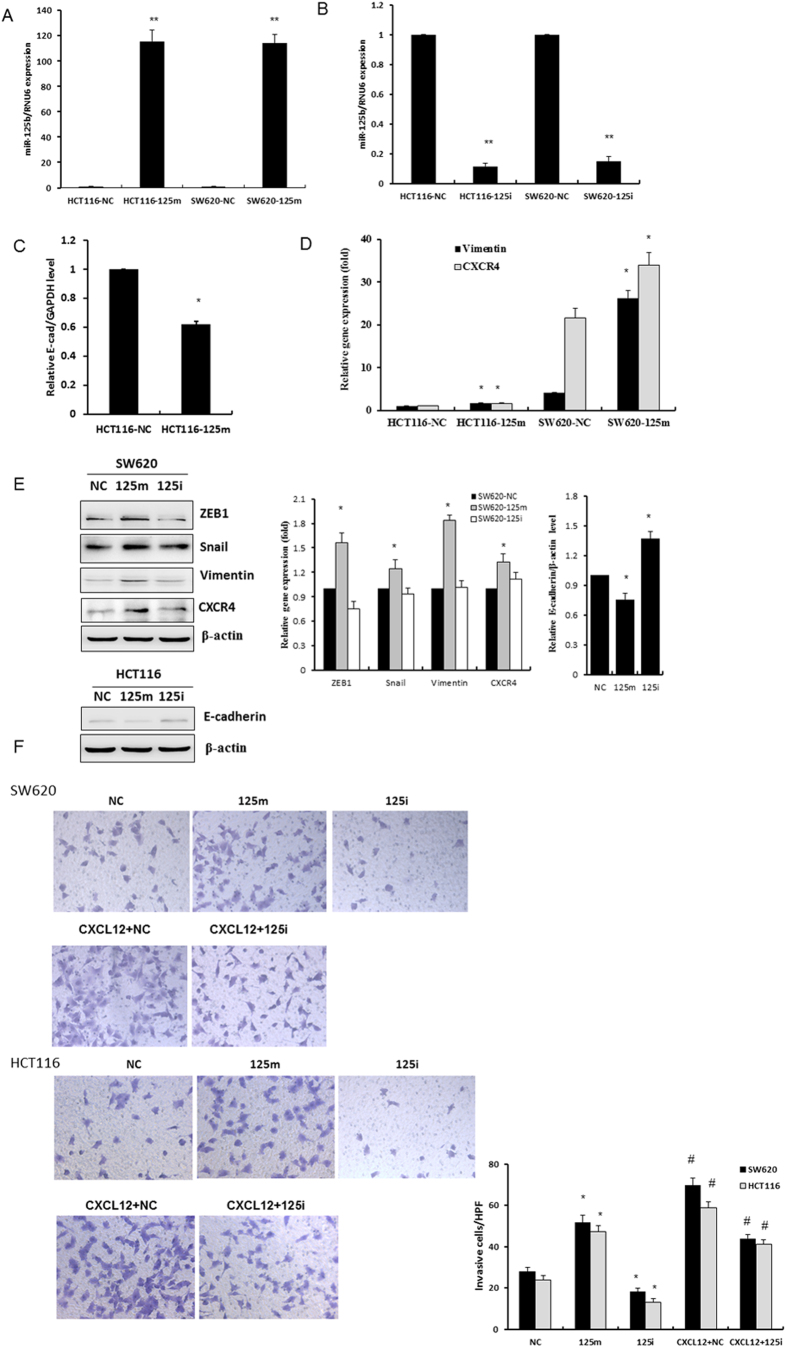
Overexpression of miR-125b promoted EMT in CRC cells. (**A**,**B**) HCT116 and SW620 cells were transfected with 100 nM miR-125b mimics (125 m) or inhibitors (125i) for 48 h. The levels of miR-125b were determined by RT-qPCR assay. (**C**,**D**) The cells were treated as indicated above, the mRNA levels of E-cadherin, vimentin and CXCR4 were determined by RT-qPCR assay. (**E**) The levels of ZEB1, Snail, vimentin and E-cadherin were determined by Western blot assay. Bar graphs indicated the relative levels of the proteins normalized to β-actin. *P < 0.05 *vs.* negative control. (**F**) HCT116 and SW620 cells were transfected with 100 nM miR-125b mimics (125 m) or inhibitor (125i) for 24 h, then were treated with or without 100 ng/μl CXCL12 for 24 h. The capacity of cell invasion was evaluated by transwell assay. ^*^P < 0.05 *vs.* negative control (NC). ^#^*P* < 0.05 vs. NC and 125i groups, respectively.

To investigate the roles of miR-125b involved in the CXCL12/CXCR4 axis induced EMT, we examined morphologic changes and expression of molecules related to EMT in cancer cells transfected with miR-125b mimics. HCT116 cells transfected with miR-125b displayed a prominent mesenchymal phenotype with an increase of spindle-shaped cells (Supplementary Fig. S1). RT-qPCR results demonstrated a significant decrease of E-cadherin in HCT116 cells (The endogenous mRNA level of E-cadherin was undetectable in SW620 cells), whereas the mRNA level of vimentin was enhanced in both HCT116 and SW620 cells (Fig. 2A–D). Additionally, transfection of miR-125b mimics into SW620 cells led to a pronounced increase of vimentin and transcriptional factor ZEB1 and Snail at the protein level. These results suggest that the increase of EMT by miR-125b is independent of the activation of CXCL12/CXCR4 axis. Consistently, miR-125b mimics suppressed, whereas miR-125b inhibitors enhanced the level of E-cadherin protein in HCT116 cells (Fig. 2E). Importantly, overexpression of miR-125b prominently increased the expression of CXCR4 at both mRNA and protein levels, thereby forming a positive feedback loop between miR-125b and activation of CXCL12/CXCR4 axis (Fig. 2E).

Transwell assay further confirmed the invasive capacities of CRC cells by regulating the expression of miR-125b. SW620 and HCT116 cells transfected with miR-125b mimics exhibited higher invasive capacity than those transfected with miR-125b inhibitors (Fig. 2F). Importantly, miR-125b inhibitors could potentially impair the invasiveness induced by CXCL12, suggesting that miR-125b was an important mediator in the progression of CRC upon the activation of CXCL12/CXCR4 axis.

### Overexpression of miR-125b activates the Wnt/β-catenin pathway by targeting APC tumor suppressor gene

**Figure 3:**
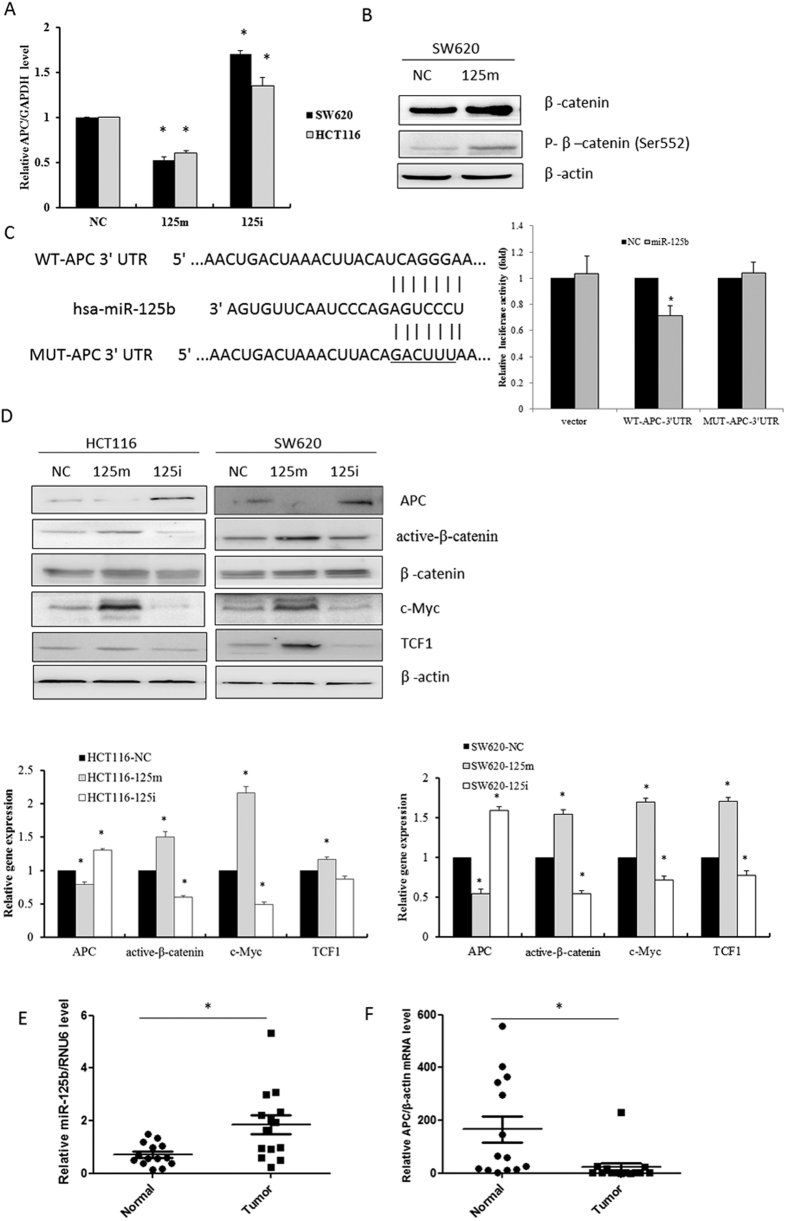
MiR-125b enhanced cell invasion by targeting *APC* gene. (**A**) HCT116 and SW620 cells were transfected with 100 nM miR-125b mimics (125 m) or inhibitors (125i) for 48 h. The mRNA level of *APC* was determined by RT-qPCR assay. (**B**) The level of p-β-catenin/β-catenin was examined by Western blot assay. (**C**) HCT116 cells were transfected with luciferase constructs and miR-125b mimics. The comparison of *luciferase* activity of wild-type (WT) and mutant (MUT) *APC*-3′UTR constructs was performed 36 h after transfection. Data was normalized to *renilla* activity. ^*^P < 0.05 *vs.* negative control (NC). (**D**) Western blotting was performed to determine the expression of APC, active-β-catenin, β-catenin, c-Myc and TCF1. Bar graphs indicated the relative levels of these proteins normalized to β-actin. ^*^P < 0.05 *vs.* negative control. (E, F) RT-qPCR was performed to examine the expression of miR-125b and *APC* mRNA in 14 pairs of CRC tissues and adjacent normal colorectal tissue. ^*^P < 0.05 *vs.* normal group.

MiR-125b plays a crucial role in the aggressiveness of CRC cells. To determine the potential targets of the oncogenic miR-125b, we predict possible target genes of miR-125b by using Targetscan programs. The results indicated that multiple genes and transcriptional factors related to cancer apoptosis and progression, such as Bak1, Bcl-2, KLF13 and STAT3, might be the target genes of miR-125b. Among these genes, we hypothesized that *APC*, the tumor suppressor gene, might be a potential target of miR-125b. HCT116 and SW620 cells transfected with miR-125b mimics impaired the expression of *APC* at both mRNA and protein levels. In contrast, miR-125b inhibitors significantly increased *APC* expression. Moreover, SW620 cells transfected with miR-125b mimics revealed an increase of p-β-catenin at the site of Ser552 (Fig. 3A,B). Phosphorylation at Ser552 significantly induces the accumulation of β-catenin in the nucleus and increases its transcriptional activity. These results indicated that miR-125b might target *APC* gene and consequently promote cancer progression. Further investigation showed that miR-125b might downregulate *APC* by targeting 3′UTR of *APC* gene as determined by dual luciferase reporter assay (Fig. 3C). Direct binding of miR-125b to the target site in the 3′UTR of *APC* was identified by cloning wild-type (WT) and mutant *APC* 3′UTR before luciferase gene in GV272 vector. The coexpression of *APC*-3′UTR constructs with miR-125b mimics in HCT116 cells resulted in an inhibition of the relative luciferase activity by approximately 30% (*P* < 0.05), as compared with scramble mimics; whereas miR-125b mimics did not inhibit the luciferase activity in the cells expressing mutant *APC*-3′UTR. These results suggested that miR-125b might directly bind to the 3′UTR of *APC* and modulate *APC* expression.

Normally, APC builds a “destruction complex” with glycogen synthase kinase 3-β (GSK-3β) and axin that is able to target β-catenin for degradation in the cytoplasm. Loss of APC functions led to dysregulation of the Wnt/β-catenin signaling pathway. Since *APC* is the potential target of miR-125b, it is possible that miR-125b could activate the Wnt/β-catenin signaling pathway through downregulation of *APC* expression. To confirm this, we transfected miR-125b mimics and inhibitors into HCT116 and SW620 cells, then examined the active form of β-catenin and a series of downstream targets of Wnt/β-catenin pathway. As expected, miR-125b overexpression enhanced the expression of active-β-catenin, TCF1, c-Myc, in both cell lines. However, downregulation of miR-125b significantly reduced the expression of these genes (Fig. 3D). TCF1 belongs to the transcriptional factor of TCF/LEF (T-cell factor/lymphoid enhancing factor) that provide docking sites for β-catenin and recruit coactivator β-catenin to the enhancer elements of target genes upon activation of Wnt signaling. c-Myc is a regulator that plays an important role in cell cycle progression and cellular transformation. The enhanced expression of these downstream genes TCF1 and c-Myc indicated activation of the Wnt/β-catenin signaling by oncogenic miR-125b.

Although many previous reports indicated the oncogenic aspects of miR-125b, there have been very few studies regarding miR-125b expression and clinical significance in CRC progression. We thus investigate the association of miR-125b with APC expression in the clinical paired CRC tissue specimens. The level of miR-125b was significantly higher while the expression of APC gene was significantly lower in CRC tissues than the adjacent normal colon tissues (*P* < 0.05, Fig. 3E,F). Indeed, there was a negative correlation between miR-125b and APC expression in CRC tissues (γ = −0.576, *P* = 0.031).

### MiR-125b confers CRC cells to 5-FU resistance through enhancing cell autophagy

**Figure 4:**
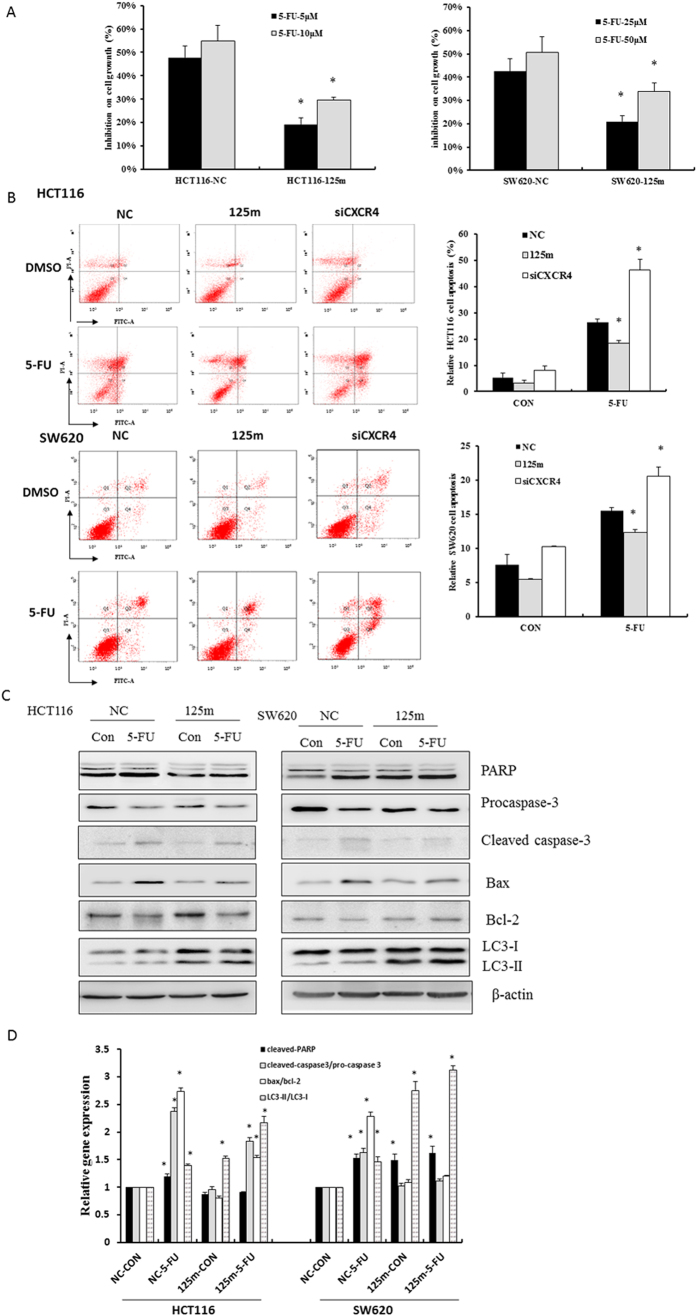
MiR-125b reduced the 5-FU-induced apoptosis in CRC cells. (**A**) HCT116 and SW620 cells were transfected with 100 nM miR-125b mimics (125 m) for 24 h, then were treated with 5, 10 μM 5-FU or 25, 50 μM 5-FU for 72 h respectively. Cell viability was estimated by the MTT assay. (**B**) HCT116 and SW620 cells were transfected with 100 nM miR-125b mimics (125 m) or 200 nM siRNA of CXCR4 for 24 h, then the cells were treated with or without 25 μM or 50 μM 5-FU for 48 h respectively. Annexin V-PI double staining assay was performed to determine apoptotic cells using flow cytometry. Bar graphs indicated the percentage of apoptotic cells. Data represent mean ± SD of three experiments. ^*^P < 0.05 *vs.* negative control (NC). (**C**) HCT116 and SW620 cells were treated as above, Western blotting was performed to analyze the expression of apoptotic proteins Bax, Bcl-2, PARP, caspase-3 and autophagic protein LC3 cleavage. Bar graphs indicated the relative levels of Bax/Bcl-2, PARP, cleaved-caspase 3/pro-caspase 3 and LC3-II/LC3-I normalized to β-actin. ^*^P < 0.05 *vs.* vehicle control, ^#^*P* < 0.05 *vs.* negative control (NC).

MiR-125b was a critical mediator of CXCL12/CXCR4 axis in cancer EMT. CXCR4 has been considered to be associated with stemness and chemoresistance. Thus, the involvement of miR-125b in the development of chemoresistance of CRC was investigated upon activation of CXCL12/CXCR4 axis. Our further findings revealed a role of miR-125b in conferring CRC cells to 5-FU resistance. HCT116 and SW620 cells transfected with miR-125b mimics and negative control were exposed to different doses of 5-FU for 72 h and then cell survival was estimated by MTT assay. The results showed that colorectal cancer cells overexpressing miR-125b exhibited lower sensitivity to 5-FU cytotoxicity than negative control. Treatment with 5 μM and 10 μM of 5-FU inhibited cell growth by 47.7 ± 4.9% and 55.0 ± 2.8% respectively when HCT116 cells were transfected with negative control miRNAs (*P* < 0.05 *vs.* vehicle control). In contrast, transfection with miR-125b mimics conferred resistance to 5-FU, showing the rates of inhibition were 19.2 ± 6.6% and 29.7 ± 3.3% respectively. Similar results were also observed in SW620 cells. There was a significant difference between cells overexpressing miR-125 and negative control in response to 5-FU treatment (*P* < 0.05, Fig. 4A).

Cancer cells transfected with miR-125b demonstrated low response to 5-FU, which was further analyzed cell apoptosis by Annexin V-PI staining assay. As shown in Fig. 4B, 5-FU treatment significantly increased apoptosis of HCT116 cells compared with vehicle control (26.3 ± 1.2% *vs.* 5.4 ± 1.6%). Cells transfected with miR-125b mimics demonstrated lower levels of apoptosis induced by 5-FU than negative control cells (18.4 ± 1.1% *vs.* 26.3 ± 1.2%, *P* < 0.05). However, knockdown of CXCR4 significantly increased the percentage of apoptotic cells up to 46.4 ± 3.9% upon 5-FU treatment (*P* < 0.05 *vs.* negative control). Similarly, overexpression of miR-125b significantly reduced the 5-FU-induced cell apoptosis in SW620 compared with negative control cells (12.3 ± 0.4% *vs.* 15.5 ± 0.5%, *P* < 0.05), whereas knockdown of CXCR4 significantly increased the level of cell apoptosis up to 20.6 ± 1.3%. These results indicated that upregulation of miR-125b as well as CXCR4 might be responsible for the development of resistance to 5-FU treatment in CRC cells.

The capability of miR-125b in reducing the sensitization of CRC cells to 5-FU induced cell apoptosis was also seen at the protein levels. 5-FU treatment significantly increased the expression of cleaved PARP as well as the ratio of Bax/Bcl-2 and cleaved caspase-3 relative to pro-caspase 3 in both HCT116 and SW620 cells (Fig. 4C). However, increased expression of these proteins was substantially attenuated in cells transfected with miR-125b mimics compared with negative control group. Since autophagy plays a prosurvival role and contributes to therapy resistance, it is plausible that miR-125b decreased the sensitivity of CRC cells to the 5-FU-induced apoptosis by induction of autophagy. We further examined the expression of specific autophagy marker LC3 in miR-125b mimics-transfected cells. LC3-II, the cleaved and lipidated form of the microtubule associated protein light chain 3 (MAP1LC3), is a hallmark protein characterizing the increase of autophagy. 5-FU-induced cleavage of LC3-Ι into LC3-II was dramatically augmented in the miR-125b mimics-transfected cells as compared with those transfected with negative control miRNA (Fig. 4C).

**Figure 5:**
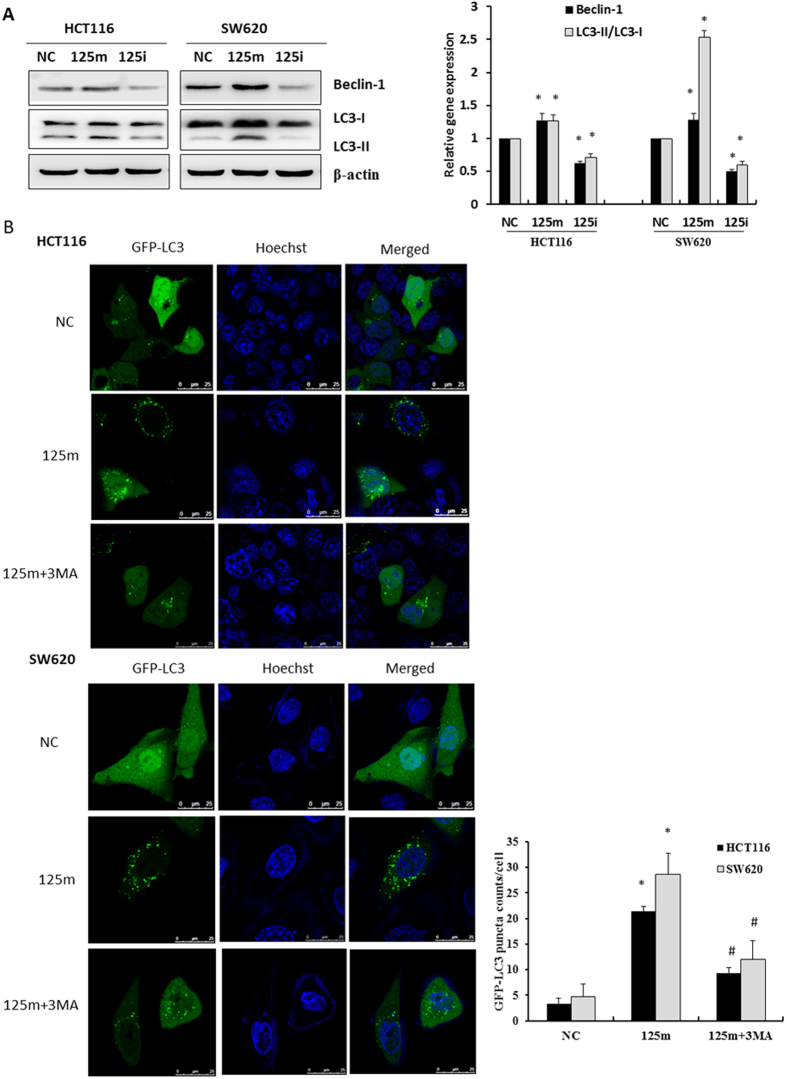
MiR-125b activated autophagic activity in CRC cells. (**A**) HCT116 and SW620 cells were transfected with 100 nM miR-125b mimics (125 m) or inhibitors (125i) for 48 h. The expression of beclin-1 and cleaved LC3-II was examined by Western blot assay. Bar graphs indicated relative levels of LC3-II and Beclin-1normalized to β-actin. Data represent mean ± SD of three experiments. ^*^P < 0.05 *vs.* negative control. (**B**) HCT116 and SW620 cells were co-transfected with GFP-LC3 plasmid and 100 nM miR-125b mimics for 48 h in the presence or absence of 5 mM 3-MA. Representative photographs were taken using a confocal microscopy (scale bar = 25 μm). Numbers of GFP-LC3 puncta per cell were counted (^*^P < 0.05 *vs.* negative control, ^#^*P* < 0.05 *vs.* 125 m group, n = 10). Data represent mean ± SD of three experiments.

To investigate the role of miR-125b in enhancing cell autophagy, we transfected miR-125b mimics and inhibitors into HCT116 and SW620 cells and analyzed the expression of beclin-1 and cleaved LC3B-II. Beclin-1 is a mammalian ortholog of the yeast autophagy-related gene 6 (Atg6) that is needed for the vesicle nucleation step of autophagy. Both of beclin-1 and LC3B-II play a critical role in the regulation of autophagy. The expression of beclin-1 and cleaved LC3B-II was significantly increased in the miR-125b mimics-transfected cells compared with negative control cells. Conversely, the cells transfected with miR-125b inhibitors exhibited reduced expression of those genes compared with negative control (Fig. 5A).

HCT116 and SW620 cells were further transfected with GFP-LC3 plasmid and then GFP-LC3-II puncta were analyzed in the autophagic cells (Fig. 5B). The number of autophagosomes (GFP-LC3-II dots) was counted in the cells transfected with miR-125b mimics in the presence or absence of autophagy inhibitor 3-Methyladenine (3-MA). The results showed that miR-125b mimics significantly increased the number of GFP-LC3 puncta. In the presence of 3-MA, the ability of autophagosomes formation was significantly suppressed (Fig. 5B, *P* < 0.05).

### MiR-125b confers the chemotherapy resistance to 5-FU in CRC xenograft expressing miR-125b *in vivo*

**Figure 6:**
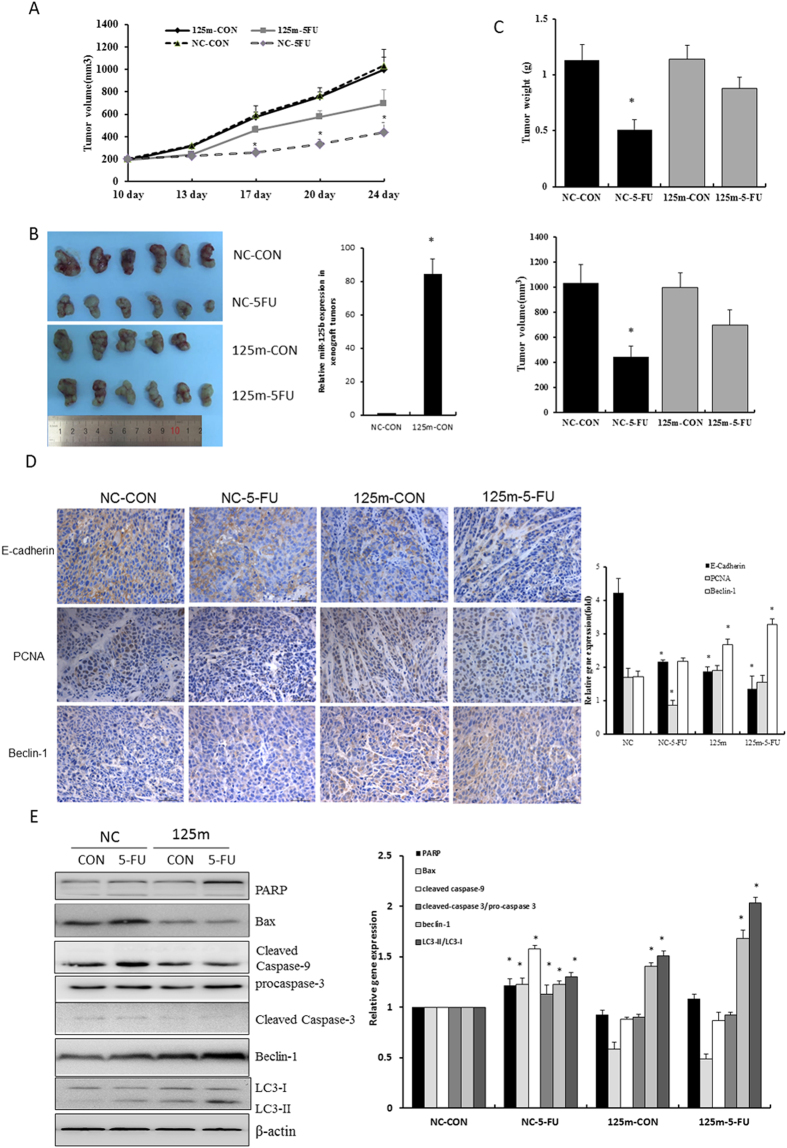
MiR-125b inhibited the 5-FU-induced apoptosis in the xenograft model *in vivo*. HCT116 cells infected with lentiviral miR-125b (125 m) and negative control (NC) were injected subcutaneously into nude mice. When tumor volume reached 100 mm^3^, 5-FU (20 mg/kg) was injected intraperitoneally five times per week for two weeks. (**A**) Tumor volumes were measured twice per week and the dynamic changes were shown in curves. Data represent mean ± SD. CON represents a short form of vehicle control. (**B**) Tumor images were taken at the end of the experiments. RNA was extracted from xenograft tumors of NC-CON and 125m-CON groups to determine the expression of miR-125b expression by RT-qPCR assay. (**C**) Tumor weight and volume were determined after removal. Data represent mean ± SD, ^*^P < 0.05 *vs.* negative control. (**D**) Representative images were shown to indicate the expression of E-cadherin, PCNA and Beclin-1 in different groups through immunohistochemistry assay(400 ×). Results were semi-quantified by image-pro plus 6 software and statistical analyses were performed. ^*^P < 0.05 *vs.* negative control. (**E**) Protein lysates were extracted from xenograft tumor tissues to determine the expression of Bax, PARP, caspases and autophagic proteins beclin-1 and LC3-II by Western blot assay. Bar graphs indicated the relative levels of cleaved PARP, cleaved caspase-9, caspase-3, LC3-II and Beclin-1 normalized to β-actin. Data represent mean ± SD of three experiments. ^*^P < 0.05 *vs.* negative control. CON represents a short form of vehicle control, 5-FU is a short form of 5-fluororacil.

HCT116 cells infected with lentiviral miR-125b and negative control miRNA were inoculated into nude mice to establish subcutaneous xenograft models. HCT116 xenografts overexpressing pre-miR-125b (125 m group) and pre-miR-NC (NC group) were exposed to 5-FU or vehicle control for two weeks. HCT116 cell implants expressing pre-miR-NC displayed higher sensitivity to 5-FU, showing a significant inhibition on cancer growth upon 5-FU exposure over time compared with vehicle control-treated group (NC-CON) (*P* < 0.05, Fig. 6A). At the end of the experiment, 5-FU significantly reduced the tumor weight and volume of NC group, whereas the effects of 5-FU did not reach statistical significance in 125 m group, although it showed a reduced trend (Fig. 6B,C, S2). Subsequently, we performed RT-qPCR assay to confirm the overexpression of miR-125b in the xenograft tumors with lentiviral miR-125b infection. Expectedly, miR-125b was highly expressed in 125 m groups (Fig. 6B). We further examined the expression of E-cadherin, proliferating cell nuclear antigen (PCNA) and Beclin-1 in the xenograft tumor tissue specimens. Immunohistochemistry results revealed that E-cadherin was predominantly expressed in tumor cell membranes and the expression level was higher in NC groups than that of 125 m groups (*P* < 0.05). Moreover, 5-FU significantly reduced E-cadherin expression in NC group while had no significant effect in 125 m group. PCNA is involved in eukaryotic DNA synthesis and plays an important role in the cell cycle regulation. Thus, it is considered as a cell proliferation marker. The results showed a typical nuclear expression of PCNA and 5-FU significantly reduced PCNA expression in NC group (*P* < 0.05). Beclin-1 was remarkably expressed in the cytoplasm of cancer cells. The expression of beclin-1 was significantly higher in 125 m group than that of NC group (*P* < 0.05). 5-FU could further potentiate the expression of beclin-1 in both NC and 125 m groups. These results indicated that overexpression of miR-125b markedly enhanced invasion, proliferation and autophagy that could contribute to the progression of colorectal carcinoma xenograft (Fig. 6D). Furthermore, 5-FU treatment led to pronounced reduction of tumor size in NC group but not in 125 m group possibly through inhibiting proliferation and inducing apoptosis of cancer cells. Further determination of the expression of apoptotic and autophagic proteins in the xenograft of tumor tissues showed that overexpressing miR-125b impaired 5-FU induced expression of apoptotic proteins Bax, cleaved caspase-3, caspase-9 and cleaved PARP. Nonetheless, it increased the 5-FU-induced autophagic proteins beclin-1 and cleaved LC3B-II determined by *in vivo* assay (Fig. 6E). These results indicated the cancer cells overexpressing miR-125b displayed less sensitivity to 5-FU than those expressing pre-miR-NC possibly due to the reduced apoptosis and enhanced autophagy.

## Discussion

The CXCL12, also termed SDF-1, is a member of the CXC family of chemokines that regulates leukocyte trafficking and is variably expressed in a number of normal and cancer tissues. CXCL12 and its receptor CXCR4 has been shown to play important roles in cancer growth, invasion and metastasis^6^,^14^. Interruption of the CXCL12/CXCR4 axis using CXCR4 inhibitor AMD3100 or neutralizing CXCR4 antibodies blocked the progression of cancer metastasis^15^. However, the underlying molecular targets involved in the activation of CXCL12/CXCR4 axis have not been fully understood.

MiRNAs have been found to regulate gene expression post-transcriptionally and play important roles in various aspects of cancer development^16^. In CRC, some miRNAs have been proposed as prognostic markers and therapeutic targets^17^,^18^. Particularly, some miRNAs have been reported to be regulated by activation of the CXCL12/CXCR4 axis^13^,^19^. In this study, we hypothesized that miRNAs might be an important mediator in the progression of cancer, such as invasion, metastasis and chemoresistance, in response to the activation of CXCL12/CXCR4 axis. The results demonstrated that human miR-125b, which positively regulates EMT and wnt/β-catenin signaling by targeting APC expression, is controlled by CXCL12/CXCR4 signaling in cancer cells. We uncovered a novel positive feedback circuit of CXCL12/CXCR4 axis and miR-125b in cancer invasion. Activation of CXCL12/CXCR4 axis led to an increase of miR-125b, which in turn enhanced the expression of CXCR4. This positive regulation of CXCR4 and miR-125b was also observed in the CXCR4 transgenic mice. CXCR4 transgenic mice exhibited a higher level of miR-125b compared with control C57B/6 J mice (Supplementary Fig. S3). Importantly, overexpression of miR-125b led to reduced sensitivity of cancer cells to the 5-FU-induced apoptosis possibly by increasing of autophagy.

MiR-125b has been reported to be dysregulated in many cancers and functioned as oncogenes in promoting growth and inhibiting apoptosis^20^,^21^. MiR-125b was found to mediate the proliferative effects of cancer through down-regulation p53^22^, pro-apoptotic Bcl-2 antagonist killer 1 (Bak1)^23^, and Bcl-2 modifying factor (Bmf)^24^. Recent studies suggested a prometastatic role of miR-125b in cancers by targeting STARD13 or TP53^25^,^26^. In this study, we found that miR-125b was upregulated in human CRC tissues compared with adjacent normal colon tissues. Further, the expression of miR-125b could be significantly induced by activation of the CXCL12/CXCR4 axis. Consistently, our results showed that miR-125b was associated with tumor invasion and was thus a prognostic marker in CRC^27^. To determine the function of miR-125b in cancers, CRC cells were transfected with miR-125b mimics or inhibitors. As expected, the results revealed that overexpression of miR-125b significantly increases cancer cell invasion and EMT, showing reduced epithelial marker E-cadherin and highly expressed mesenchymal marker vementin.

MiRNAs are small noncoding regulatory RNAs that potentially target a large number of genes. To investigate the targets of miR-125b, we used Targetscan program to predict the potential targets. Our initial results showed that Adenomatous polyposis coli (*APC*) mRNA and protein were downregulated by transfection with miR-125b mimics. Further luciferase assay revealed that miR-125b could directly bind to 3′UTR of *APC* gene. *APC* is thus one of the potential targets of miR-125b. *APC* functions as an important tumor suppressor gene that antagonizes Wnt/β-catenin signaling pathway^28^. APC mutation results in activation of the Wnt/β-catenin signaling pathway and thus initiates colonic tumorigenesis. Our results demonstrated that miR-125b played an important role in triggering tumor invasion through activation of the Wnt/β-catenin signal pathway by targeting *APC* gene. Wnt/β-catenin signaling functions in promoting epithelial-mesenchymal transition (EMT) phenotype^29^. Moreover, Wnt/β-catenin signaling is considered to be a potential regulator of stemness and chemoresistance in colon cancers^30^,^31^. Indeed, activation of the Wnt/β-catenin signaling induced by upregulation of miR-125b is strongly correlated with the progression and stemness of CRC^32^.

5-Fluorouracil (5-FU) is widely used in the treatment of many cancers^33^. However, the clinical efficacy of 5-FU is limited due to development of chemoresistance^34^. Although various reasons have been investigated, the underlying mechanisms have not been fully understood. Accumulating evidences have indicated a prominent role of miR-125b in conferring resistance to chemotherapy^23^,^35^,^36^. In this study, we shed a new light on the role of miR-125b in the mechanism of cancer resistance to 5-FU therapy through increasing autophagy. In most cases, autophagy might protect cancer cells from the unfavorable growth condition and thus attenuate the efficiency of most anticancer drugs^37^,^38^. In this study, miR-125b was found to confer 5-FU resistance by increasing autophagy, showing enhancement of beclin-1 and cleaved LC3-II as well as increased formation of autophagosomes (GFP-LC3 dots). Similar results were further observed in the miR-125b overexpressing HCT116 xenograft tumor model.

In summary, we shed novel insights into the role of miR-125b in the invasion and 5-FU resistance in CRC. MiR-125b was found to promote EMT and activation of Wnt/β-catenin signaling by targeting APC. Importantly, miR-125b was upregulated by the activation of CXCL12/CXCR4 axis, and then miR-125b in turn enhanced the expression of CXCR4, which forms a positive feedback loop in triggering tumor invasion and progression. These results shed a new light on the role of miR-125b in the progression of CRC and development of chemoresistance, which provided potential therapeutic targets for treatment of CRC.

## Materials and Methods

### Cell lines and cell viability assay

Human colorectal adenocarcinoma cell lines SW620 and HCT116 were purchased from American Type Culture Collection (ATCC, Rockville, MD). Cells were grown in RPMI 1640 medium containing 10% fetal bovine serum (FBS) and penicillin/streptomycin at 37 °C in a humid atmosphere (5% CO_2_). Cell viability was estimated by the 3-[4,5-dimethylthiazol-2-yl]−2,5-diphenyltetrazolium bromide (MTT) assay.

### Lentivirus infection and cell transfection

Cancer cells were infected with lentivirus expressing CXCR4 (LV-CXCR4) or empty vector GV287 sequences under control of Ubi promoter synthesized by Genechem (Shanghai, China). The lentiviral supernatants were used to infect cells in the presence of polybrene at 10 μg/ml for 12 h. Cells were then cultured in fresh media containing 2 μg/ml of puromycin to obtain stable cell line. To knockdown CXCR4, 200 nM of siRNA-1 and siRNA-2 with oligonucleotide sequences 5′-UCCUGGCCUUCAUCAGUCUTT-3′ and 5′-GCGAGGUGGACAUUCAUCUTT-3′ were used to transfect cells for 48 h (GenePharma, Shanghai, China). To determine the roles of MiR-125b in the activation of CXCL12/CXCR4 axis, cells were transfected with 100 nM of miR-125b mimics or miR-125b inhibitor (GenePharma Shanghai, China) for 48 h by using lipofectamine 2000 (Invitrogen) according to manufacturer’s instructions.

### RNA extraction and RT-qPCR

Total RNA was extracted by using TRIzol reagent (Invitrogen). Reverse transcription of total miRNA was performed by using a miScript reverse transcription kit (Qiagen) in accordance with manufacturer’s protocol. MiScript SYBR Green PCR kit (Qiagen) and a pair of miR-125b specific primers were used to detect mature miRNA. RNU6B was used as an internal control.

To determine mRNA expression, total RNA was reversely transcribed using SuperScript III First-Strand Synthesis System (Invitrogen) according to manufacturer’s instruction. Real-time PCR was performed in triplicate in an ABI 7500 fast real-time PCR system (Applied Biosystems) with Brilliant II SYBR Green qPCR master mix and gene-specific primers. Primer sequences for human CXCR4 were 5′-ggtggtctatgttggcgtct-3′ and 5′-tggagtgtgacagcttggag-3′; E-cadherin primers were 5′-acgcattgccacatacactc-3′ and 5′-agaggttcctggaagagcac-3′; vimentin primers were 5′-agatggcccttgacattgag-3′ and 5′-ccagagggagtgaatccaga-3′ ; APC primers were 5′-cctagaaccaaatccagcag-3′ and 5′-acatgagtggggtctcctg-3′. Primer sequences for GAPDH were 5′-gagtcaacggatttggtcgt-3′ and 5′-ttgattttggagggatctcg-3′. The thermal cycling was initiated by polymerase activation step for 10 min at 95 °C followed by 40 cycles of denaturation (95 °C for 30 s) and annealing/extension (60 °C for 1 min). Target gene expression levels were normalized to GAPDH as an housekeeping control and determined by a previously described method^39^.

### Western blotting analysis

Cells were harvested and lysed in the radioimmune precipitation assay (RIPA) buffer. Protein concentration was determined by using a BCA kit (Thermo Fisher Scientific) according to manufacturer’s instruction. Equal amounts of cell lysates were resolved by SDS-PAGE and transferred to PVDF membranes (Millipore). After blocking in 5% nonfat milk for 1 h, the membranes were immunoblotted overnight at 4 °C with primary antibodies against E-cadherin, Vimentin, Snail, ZEB1, LC3, Beclin-1, Caspase-9, Caspase-3, Bcl-2, PARP, APC, β-catenin, p-β-catenin, active-β-catenin, c-Myc, TCF1 (Cell Signaling Technology, USA), β-actin and Bax (Santa Cruz, USA) followed by incubation with polyclonal HRP-conjugated secondary antibodies for 1 h at room temperature. Immunoreactive products were visualized using Fluorchem FC3 system (Proteinsimple, USA) by chemiluminescence (Millipore, USA) and quantified by densitometry using Alphaview software. Densitometric analyses of bands were normalized with β-actin functioning as a loading control.

### Annexin V-FITC/PI staining assay

Cancer cells were transfected with 100 nM miR-125b mimics or 200 nM CXCR4 siRNA and corresponding negative control for 24 h. Cells were then treated with 25 or 50 μM 5-FU for 48 h. Levels of phosphatidylserine on cell surface were quantitatively determined by using Annexin V-fluorescein isothiocyanate (FITC) and Propidium Iodide (PI) apoptosis detection kit according to manufacturer’s instruction (Biosciences). Apoptotic cells were estimated in a flow cytometer (BD Biosciences, USA). Results were expressed as percentage of apoptotic cells relative to total cells.

### Cell autophagy analysis

Cells were transfected with GFP-LC3 plasmid (Addgene) and 100 nM miR-125b mimics in the presence or absence of 3MA for 48 h. GFP-LC3-II-positive punctate pattern was observed under confocal microscope (Leica TCS SP5) equipped with oil immersion lens (63x magnification) with 405- and 488-nm excitation lasers. Number of autophagosomes was counted by using Image J program (National Institutes of Health).

### Cell invasion assay

Cell invasion assay was performed with 24-well invasion assay kit (Corning, NY, USA). The transwell membrane was coated with a 300 ng/μl Matrigel basement membrane matrix (Corning, USA). 3 × 10^4^ cells resuspended in serum-free medium were added to the interior of inserts, 600 μl medium containing 10% FBS was added to the lower chamber as chemoattractant. Cells were incubated for 24 h at 37 °C in a CO_2_ incubator (5% CO_2_). After incubation, the upper surface of chamber was scraped to remove noninvasive cells. Transwell membrane was fixed with methanol and stained with 0.4% crystal violet to be photographed under a light microscope. Number of invaded cells per chamber was counted in five fields and average values were calculated.

### Plasmid construction and luciferase reporter assay

3′ UTR of *APC* gene was amplified using the primers with sequences were 5′- GATCGCCGTGTAATTCTAGATTAATTATTGCTTGTCTTAAAATAATG-3′ and 5′- CCGGCCGCCCCGACTCTAGAGAACCATATTACTGTGAATTTC-3′. PCR was performed with DNA containing miR-125b binding sites and digested by *Xba* I and cloned into GV272 vector (GeneChem, Shanghai). Wild type and mutant inserts were confirmed by sequencing.

For luciferase reporter assay, HCT116 cells seeded in a 96-well plate were co-transfected with firefly luciferase constructs (100 ng) and 40 nM miR-125b mimics using lipofectamine 2000 reagent. Cells were also transfected with 5 ng of pRL-SV40 plasmid to monitor transfection efficiency. *Firefly* luciferase activity was evaluated by using a dual-luciferase reporter assay system (Promega). *Renilla* luciferase activity was evaluated to normalize firefly luciferase activity for each sample. Transfections were performed in triplicate and the experiments were repeated twice.

### Human CRC tissue specimens

The experiment was approved by the Institutional Review Board of Shandong Tumor Hospital (201502005), and written informed consents were obtained from all patients. All methods with relation to human tissue were performed in accordance with the relevant guidelines and regulations by including statements in the methods section. Fourteen pairs of CRC tissue specimens and adjacent normal mucosa were obtained from Shandong Tumor Hospital (Jinan, China). Each pair of cancer specimen and adjacent normal tissue was confirmed by pathological analysis. CRC tissues were immediately frozen in liquid nitrogen for RT-qPCR analysis.

### Subcutaneous xenograft assay

Animal experiments were approved by Animal Welfare Committee of Capital Medical University (AEEI-2014-101). All methods were performed in accordance with the relevant guidelines and regulations by including a statement in the methods section. Female Balb/c athymic (nu/nu) mice, 6 weeks of age, were purchased from Vital River Laboratory Animal Center (Beijing, China). Tumors were produced by inoculating HCT116 cells transfected with pre-miR-125b or pre-miR-NC (2 × 10^6^ in 200 μl serum-free medium per mouse) subcutaneously into the armpit of a mouse. Three weeks later, the exuberantly proliferating HCT-116 overexpressing either miR-125b or miR-NC xenografted tumor tissues were cut into 1.5 mm thick pieces and inoculated subcutaneously into the armpit of mice with a puncture needle. When tumor volumes reached approximately 100 mm^3^, mice bearing the xenografts expressing miR-125b or miR-NC were randomly divided into the vehicle control group (n = 6, N.S by intraperitoneal (i.p.) injection) and 5-FU group (n = 6, 20 mg/kg by i.p. injection). Mice were observed daily for any symptoms. Administrations were performed five times per week for two weeks. Tumor growth was examined twice per week using calipers and tumor volumes were calculated using the formula volume (mm^3^) = L × W^2^/2 (length L, mm; width W, mm). Specimens of HCT-116 xenografts were removed for further analysis.

### Immunohistochemistry

Tissues were fixed in 4% paraformaldehyde and subsequently embedded in paraffin. Immunohistochemistry was performed on 5 μm paraffin embedded sections from xenograft colorectal carcinoma. Paraffin embedded sections were deparaffinized in xylene and rehydrated in a graded alcohol series and water. For Immunohistochemical Examination, the slides were heated in antigen retrieval solution, blocked in 5% bovine serum albumin (diluted with PBS) and incubated with anti-E-cadhrin (1:200), anti-PCNA (1:500) and anti-Beclin-1 (1:100) antibodies overnight at 4 °C. The sections were subsequently incubated with secondary antibodies (1:200) for 50 min at room temperature, washed with PBS and then developed with 3,3′-diaminobenzidine. Finally, the slides were counterstained with hematoxylin and mounted. An optical microscope (Leica DM6000B, × 400) was used to observe the samples. The results were semi-quantified by image-pro plus 6 software (IPP, USA).

### Statistical analysis

All data were presented as mean ± SD and statistical data were analyzed using SPSS 12.0. Statistical differences among multiple groups were evaluated by one-way analysis of variance (ANOVA) followed by Dunnett (multiple comparisons to the same control) post hoc tests. Student’s *t* test was used to compare differences between two groups. The Spearman’s correlation was used to evaluate potential correlations between miR-125b and *APC* gene expression in paired CRC tissues. Values of *P* < 0.05 were considered as statistically significant.

## Additional Information

**How to cite this article**: Yu, X. *et al*. CXCL12/CXCR4 axis induced miR-125b promotes invasion and confers 5-fluorouracil resistance through enhancing autophagy in colorectal cancer. *Sci. Rep.* **7**, 42226; doi: 10.1038/srep42226 (2017).

**Publisher's note:** Springer Nature remains neutral with regard to jurisdictional claims in published maps and institutional affiliations.

## Supplementary Material

Supplementary Information
