# Supplementary material for: CXCL12/CXCR4 axis induced miR-125b promotes invasion and confers 5-fluorouracil resistance through enhancing autophagy in colorectal cancer
Source: Sci Rep. 2017 Feb 8;7:42226. doi: 10.1038/srep42226 (PMC5296742; doi:10.1038/srep42226)
Supplement: Supplementary Information [file srep42226-s1.pdf]

CXCL12/CXCR4 axis induced miR-125b promotes invasion and confers 5-fluorouracil resistance through enhancing autophagy in colorectal cancer

Xinfeng Yu[1], Wenna Shi[1], Yuhang Zhang[1], Xiaohui Wang[2], Shiyue Sun[1], Zhiyu Song[1], Man Liu[1], Qiao Zeng[1], Shuxiang Cui[3], Xianjun Qu[1*]

[1]Department of Pharmacology, School of Basic Medical Sciences, Capital Medical University, Beijing, China

[2]Department of General Surgery, Xuan Wu Hospital, Capital Medical University, Beijing, China

[3]Beijing Key Laboratory of Environmental Toxicology, Department of Toxicology and Sanitary Chemistry, School of Public Health, Capital Medical University, Beijing, China

*Correspondence: Xianjun Qu, Department of Pharmacology, School of Basic Medical Sciences, Capital Medical University, Beijing, China

Address: No.10, You An Men Wai Avenue, Beijing, 100069

Tel/Fax: 86-10-83911516

E-mail: quxj@ccmu.edu.cn

Supplementary information

Fig. S1

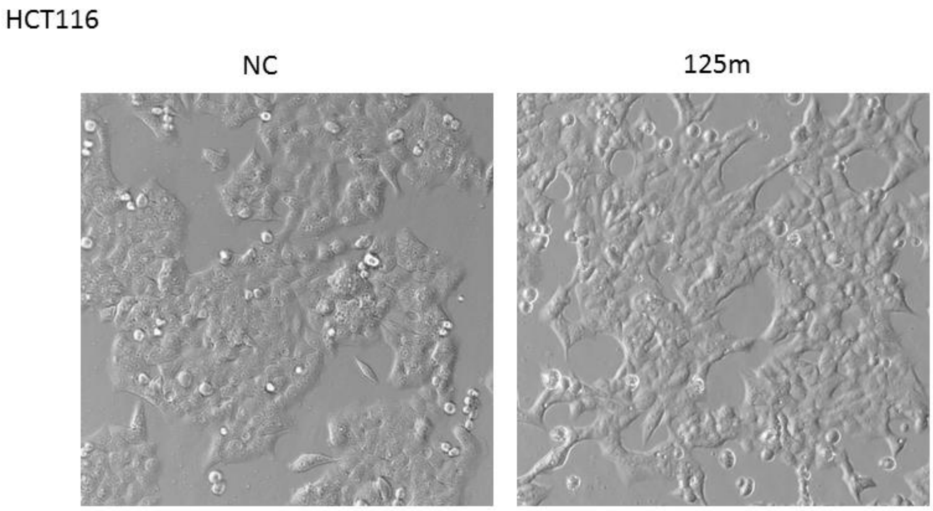

Fig. S1 Overexpression of miR-125b enhanced the mesenchymal phenotype of HCT116 cells. Cells were transfected with 100 nM miR-125b mimics and negative control miRNA for 48 h. Cell images were taken using inverted optical microscope (Nikon, Japan) to observe the changes of cellular morphology.

Fig.S2

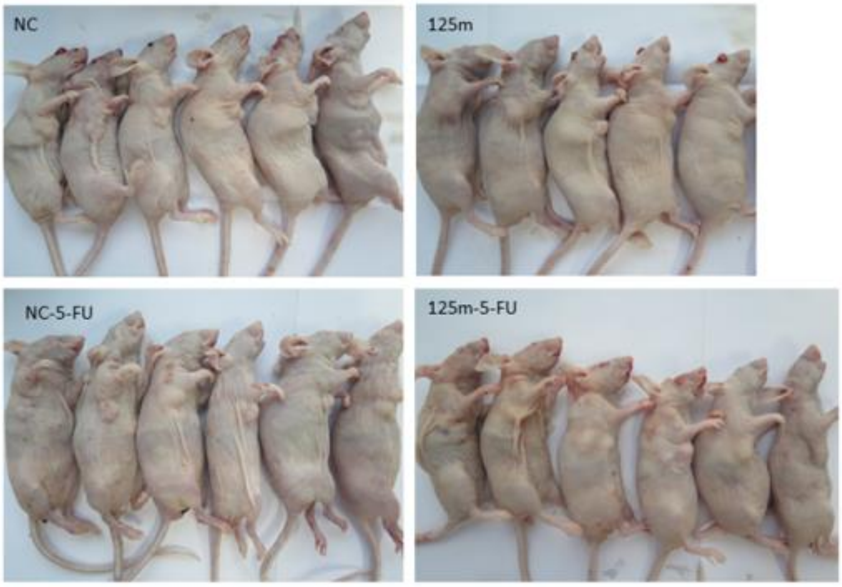

Fig. S2 The photos were taken in nude mice bearing tumors.

Fig. S3

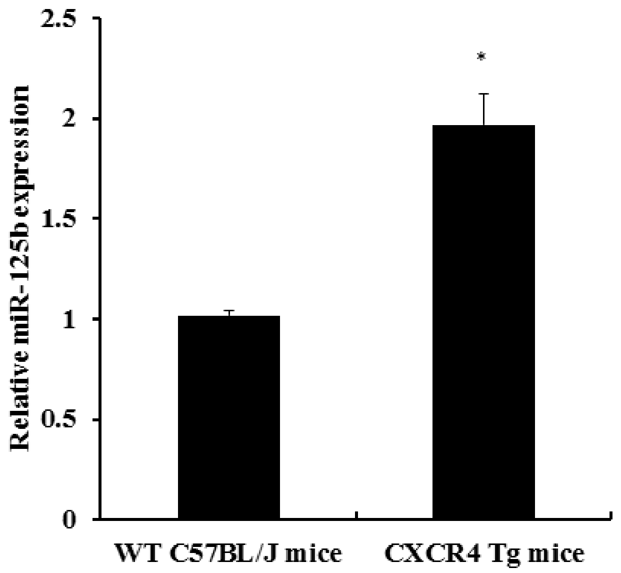

Fig. S3 The expression of miR-125b was significantly increased in CXCR4 transgenic (CXCR4 Tg) mice compared with wild-type C57BL/6J mice. RT-qPCR analysis was performed in colon tissues of CXCR4 Tg and wild-type C57BL/6J mice to determine the level of miR-125b. Values are expressed as mean±SD. * $P < 0.05$ compared with the control group.

Table 1. Differentially expressed miRNAs in HCT116 cells overexpressing CXCR4

miRNA	CXCR4_Normalize	CON Normalize	log2FoldChange	*P* value	Up/Down
hsa-miR-496	1.89	0.02	4.23	0.025	up
hsa-miR-584-3p	3.40	0.40	3.08	0.006	up
hsa-miR-379-5p	4.15	0.60	2.78	0.003	up
hsa-miR-219a-5p	2.64	0.40	2.72	0.027	up
hsa-miR-1268b	41.15	6.84	2.59	0.000	up
hsa-miR-483-5p	8.68	2.01	2.11	0.000	up
hsa-miR-133a-3p	5.66	1.41	2.01	0.005	up
hsa-miR-3138	6.42	1.61	2.00	0.003	up
hsa-miR-3165	3.77	1.01	1.91	0.034	up
hsa-miR-3126-5p	4.91	1.41	1.80	0.018	up
hsa-miR-6511b-3p	4.15	1.21	1.78	0.033	up
hsa-miR-16-1-3p	6.79	2.01	1.76	0.005	up
hsa-miR-301b-5p	4.91	1.61	1.61	0.031	up
hsa-miR-491-3p	11.70	4.02	1.54	0.001	up
hsa-miR-3127-5p	9.06	3.22	1.49	0.004	up
hsa-miR-503-5p	13.21	5.43	1.28	0.002	up
hsa-miR-374b-5p	730.05	315.96	1.21	0.000	up
hsa-let-7b-5p	4632.85	2008.18	1.21	0.000	up
hsa-miR-196b-3p	18.87	8.25	1.19	0.000	up
hsa-miR-96-3p	7.17	3.22	1.16	0.046	up
hsa-miR-483-3p	14.72	6.64	1.15	0.002	up
hsa-miR-664a-3p	25.67	11.66	1.14	0.000	up
hsa-miR-92b-5p	20.38	9.45	1.11	0.000	up
hsa-miR-197-3p	179.30	83.67	1.10	0.000	up
hsa-miR-362-3p	15.10	7.24	1.06	0.004	up
hsa-miR-624-3p	19.63	9.65	1.02	0.001	up
hsa-miR-125b-5p	5969.90	2946.72	1.02	0.000	up
hsa-miR-149-5p	757.61	377.30	1.01	0.000	up
hsa-miR-93-3p	73.23	156.07	-1.09	0.000	down
hsa-miR-222-3p	31473.42	69628.03	-1.15	0.000	down
hsa-miR-130b-5p	178.17	404.45	-1.18	0.000	down
hsa-miR-487b-3p	3.40	12.87	-1.92	0.000	down
hsa-miR-676-3p	1.51	6.44	-2.09	0.002	down
hsa-miR-6514-5p	0.38	2.41	-2.68	0.038	down
hsa-miR-369-5p	0.38	3.42	-3.18	0.005	down
hsa-miR-653-3p	0.38	3.42	-3.18	0.005	down
hsa-miR-4473	0.19	2.21	-3.55	0.021	down
hsa-miR-3129-5p	0.19	2.61	-3.79	0.009	down

Supplementary methods

1. Transgenic mice

CXCR4 transgenic mice ($CXCR4^{Tg}$) were created by Cyagen Biosciences Inc. (Guangzhou, China). In brief, transgenic founder mice were generated on a C57BL/6J genetic background. The mVillin promoter (a fragment approximate 12.7kb) on the BAC clone RP23-5K8 was transferred to the intermediate vector through gap-repair cloning method. The Cxcr4-IRES-EGFP was cloned into pStart-K plasmid to construct the intermediate vector. The pStart-K-*mVillin* promoter-Cxcr4-IRES-EGFP plasmid was purified for microinjection. The eggs were microinjected with the DNA solution under a microscope. The injected fertilized eggs were transplanted into the oviducts of pseudo-pregnant C57BL/6J mice. The positive transgenic founder mice were bred with C57BL/6J mice to generate the transgenicmice. Genotyping was performed by polymerase chain reaction with following primers: forward 5'-CTTCTCCTCTAGGCTCGTCCAC-3' and reverse 5'-GATCACCAATCCATTGCCGACT-3'. Animal experiments were approved by Animal Welfare Committee of Capital Medical University (AEEI-2014-101).

2. Sequencing analysis

Total RNA was extracted from HCT116 cells infected with lentiviral CXCR4 (Genechem, Shanghai) and negative control using Trizol reagent (Invitrogen) according to manufacturer’s protocols. RNA integrity and concentration was assessed by using the RNA Nano 6000 Assay Kit of the Bioanalyzer 2100 system (Agilent, CA). Library construction and sequencing were performed on an Illumina HiSeq2500

sequencer according to manufacturer's specifications (Illumina) at AnnoroadGene Technology (Beijing, China). Small RNA reads were filtered to get clean data and known miRNAs identification refers to miRBase 21.0 (http://www.mirbase.org). The remaining reads were used to predict novel miRNAs using software miRDeep2. DEGseq v1.18.0 was used for differential gene expression analysis between two samples. miRNAs with $P < 0.05$ and $|log2\_ratio| \geq 1$ are identified as differentially expressed miRNAs.

Fig.1

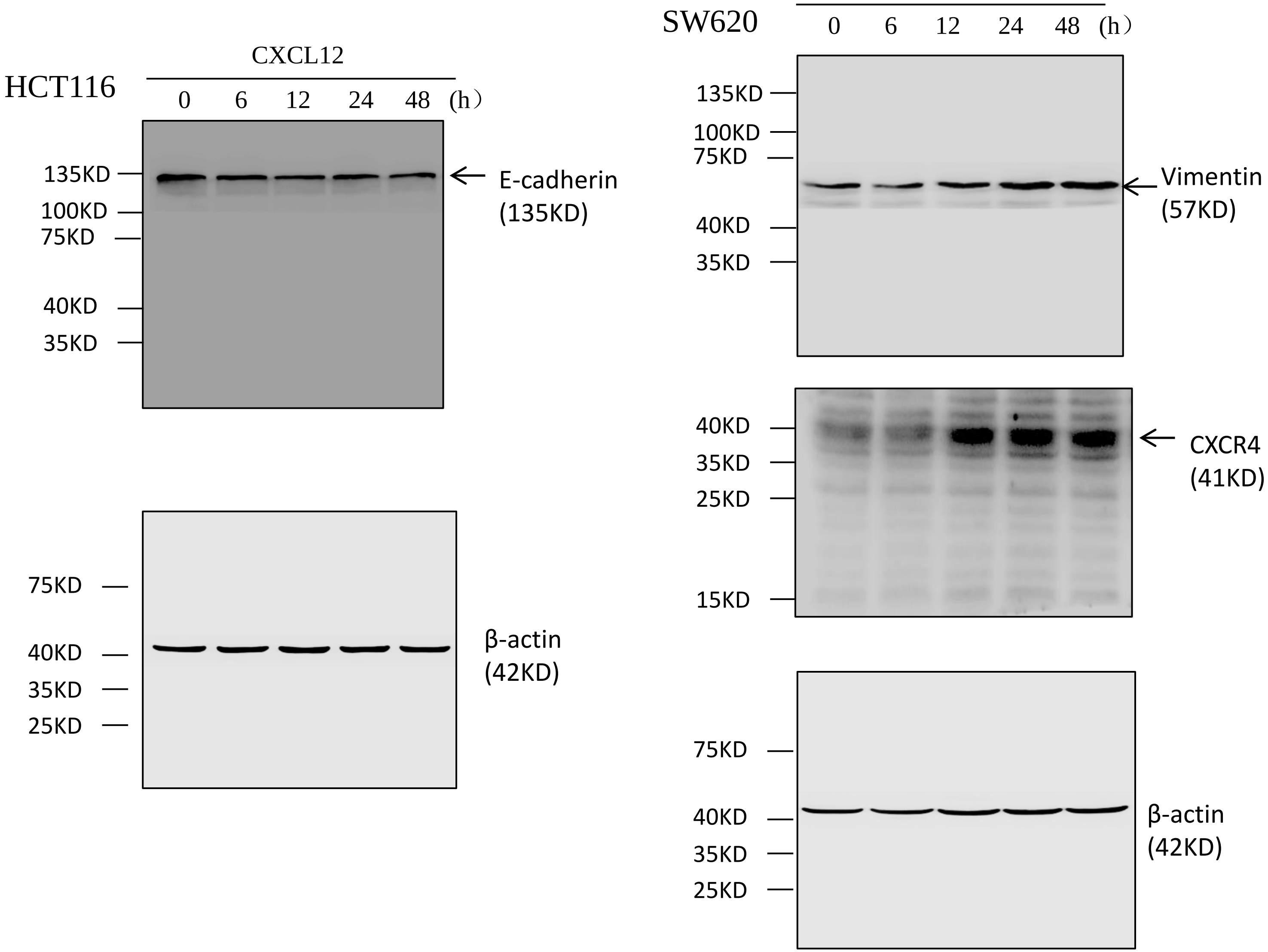

HCT116
CXCL12
0
6
12
24
48
(h）
135KD
100KD
75KD
40KD
35KD
E-cadherin
(135KD)
75KD
40KD
35KD
25KD
β-actin
(42KD)
SW620
CXCL12
0
6
12
24
48
(h）
135KD
100KD
75KD
40KD
35KD
Vimentin
(57KD)
40KD
35KD
25KD
15KD
CXCR4
(41KD)
75KD
40KD
35KD
25KD
β-actin
(42KD)

Fig.2

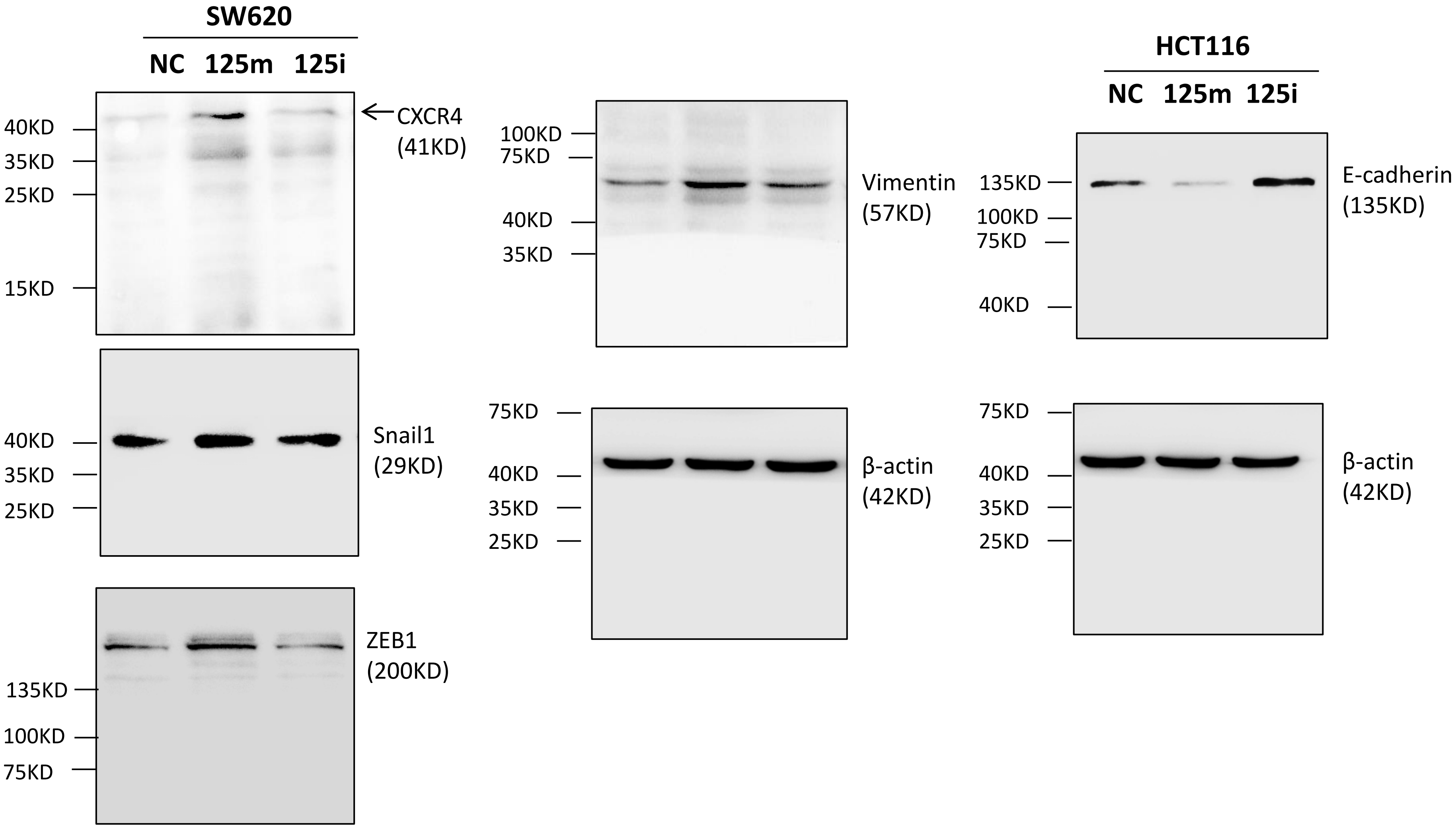
SW620
NC
125m
125i
CXCR4
(41KD)
40KD
35KD
25KD
15KD
Snail1
(29KD)
40KD
35KD
25KD
ZEB1
(200KD)
135KD
100KD
75KD
100KD
75KD
40KD
35KD
Vimentin
(57KD)
75KD
40KD
35KD
25KD
β-actin
(42KD)
HCT116
NC
125m
125i
E-cadherin
(135KD)
135KD
100KD
75KD
40KD
75KD
40KD
35KD
25KD
β-actin
(42KD)

Fig. 3

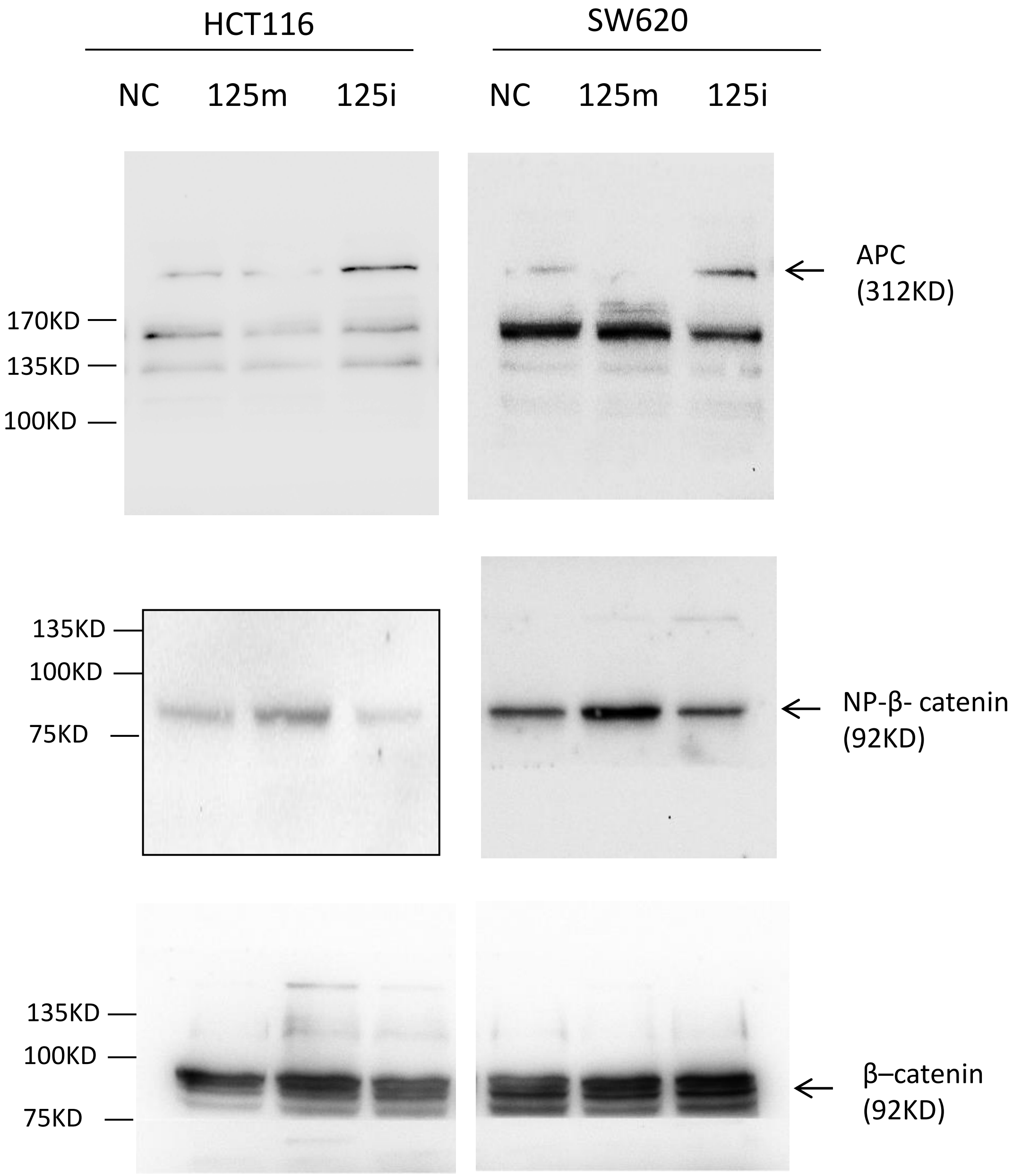
HCT116
SW620
NC
125m
125i
NC
125m
125i
170KD
135KD
100KD
APC
(312KD)
135KD
100KD
75KD
NP-β- catenin
(92KD)
135KD
100KD
75KD
β–catenin
(92KD)

Fig. 3

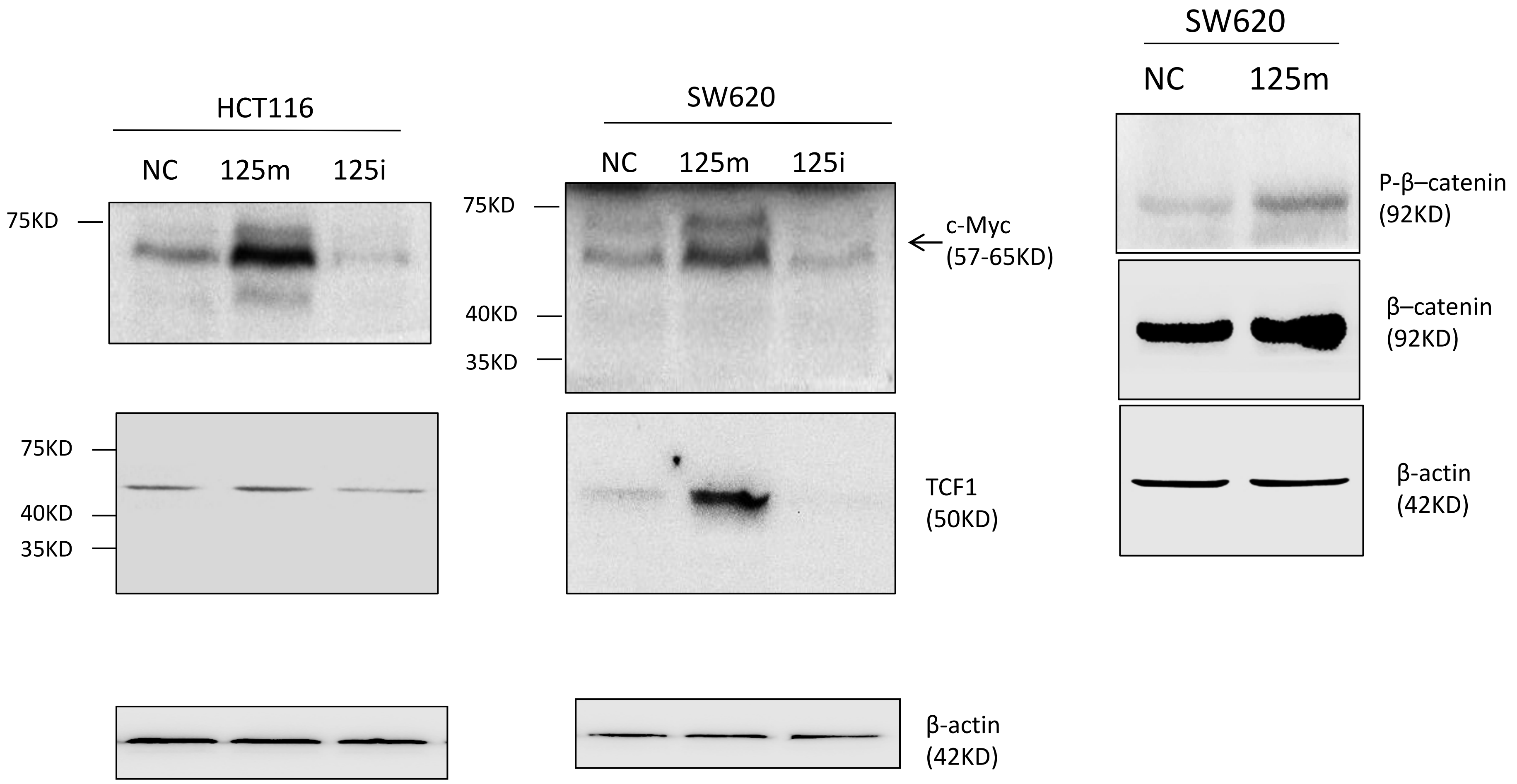
HCT116
NC
125m
125i
75KD
75KD
40KD
35KD
SW620
NC
125m
125i
75KD
40KD
35KD
c-Myc
(57-65KD)
TCF1
(50KD)
β-actin
(42KD)
SW620
NC
125m
P-β–catenin
(92KD)
β–catenin
(92KD)
β-actin
(42KD)

Fig. 4

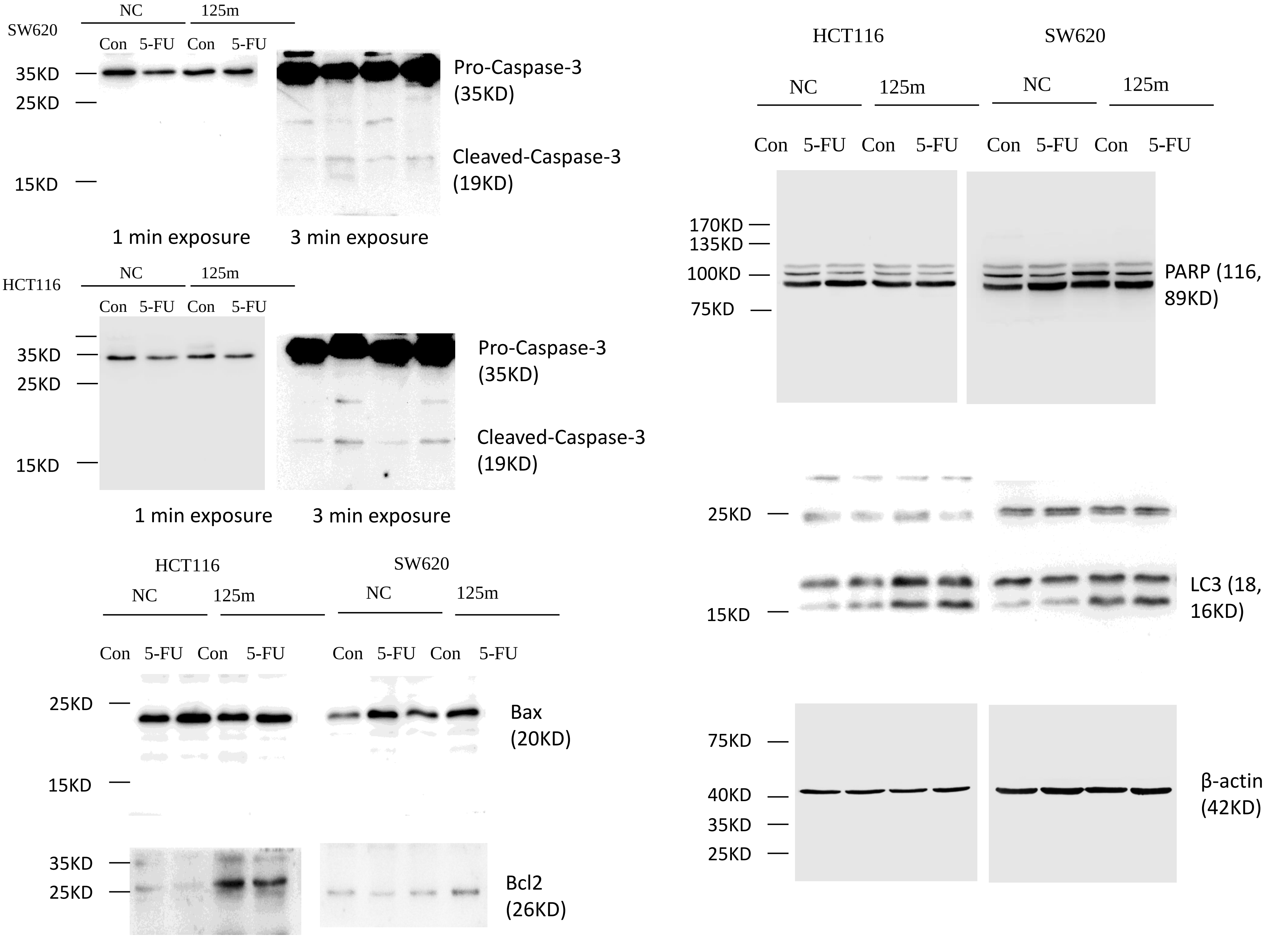
SW620
NC
125m
Con
5-FU
Con
5-FU
35KD
25KD
15KD
Pro-Caspase-3
(35KD)
Cleaved-Caspase-3
(19KD)
1 min exposure
3 min exposure
HCT116
NC
125m
Con
5-FU
Con
5-FU
35KD
25KD
15KD
Pro-Caspase-3
(35KD)
Cleaved-Caspase-3
(19KD)
1 min exposure
3 min exposure
HCT116
SW620
NC
125m
NC
125m
Con
5-FU
Con
5-FU
Con
5-FU
Con
5-FU
25KD
15KD
Bax
(20KD)
35KD
25KD
Bcl2
(26KD)
HCT116
SW620
NC
125m
NC
125m
Con
5-FU
Con
5-FU
Con
5-FU
Con
5-FU
170KD
135KD
100KD
75KD
PARP (116,
89KD)
25KD
15KD
LC3 (18,
16KD)
75KD
40KD
35KD
25KD
β-actin
(42KD)

Fig.5

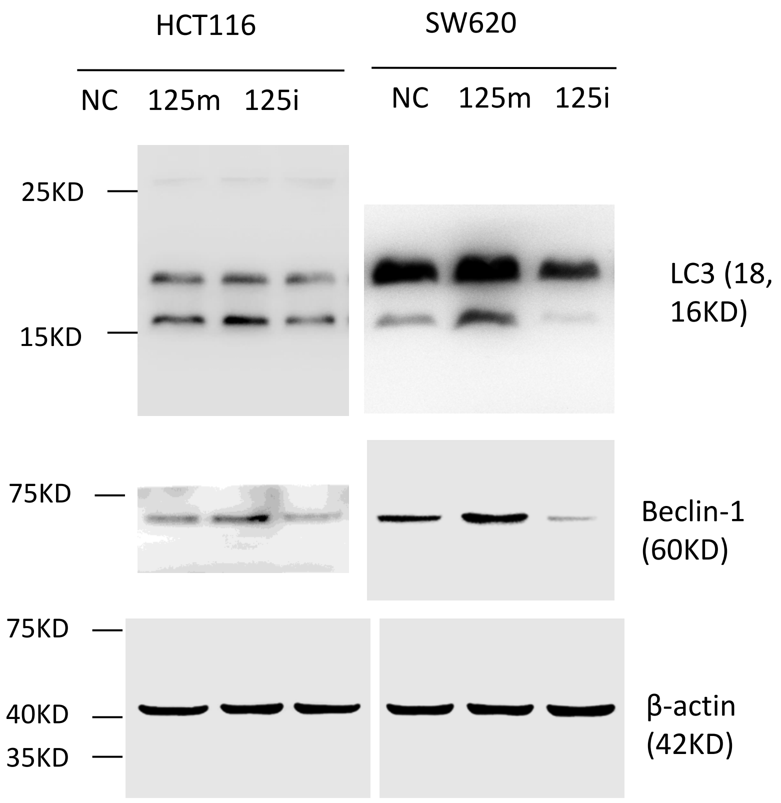
HCT116
SW620
NC
125m
125i
NC
125m
125i
25KD
15KD
LC3 (18, 16KD)
75KD
Beclin-1 (60KD)
75KD
40KD
35KD
β-actin (42KD)

Fig.6

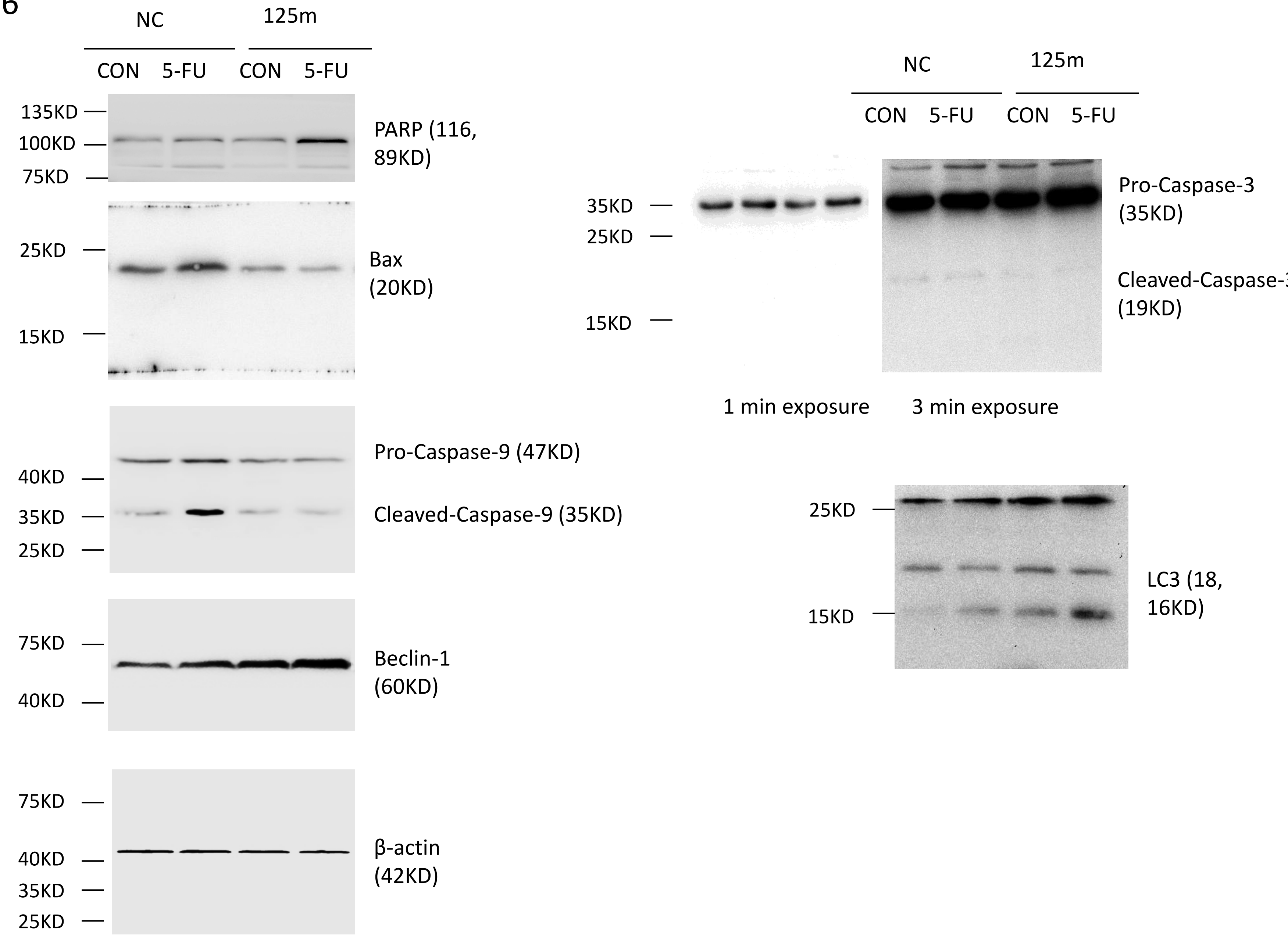
NC
125m
CON
5-FU
CON
5-FU
135KD
100KD
75KD
PARP (116, 89KD)
25KD
15KD
Bax (20KD)
Pro-Caspase-9 (47KD)
40KD
35KD
25KD
Cleaved-Caspase-9 (35KD)
75KD
40KD
Beclin-1 (60KD)
75KD
40KD
35KD
25KD
β-actin (42KD)
NC
125m
CON
5-FU
CON
5-FU
35KD
25KD
15KD
Pro-Caspase-3 (35KD)
Cleaved-Caspase-3 (19KD)
1 min exposure
3 min exposure
25KD
15KD
LC3 (18, 16KD)
